# Influences on recruitment to randomised controlled trials in mental health settings in England: a national cross-sectional survey of researchers working for the Mental Health Research Network

**DOI:** 10.1186/1471-2288-14-23

**Published:** 2014-02-17

**Authors:** Rohan Borschmann, Sue Patterson, Dilkushi Poovendran, Danielle Wilson, Tim Weaver

**Affiliations:** 1 Institute of Psychiatry, King’s College London, London, United Kingdom; 2 Metro North Hospital and Health Service, Queensland Health, Brisbane, Australia; 3 Imperial College London, London, United Kingdom

**Keywords:** Randomised controlled trials, Recruitment, Mental health, Survey, Clinical studies officers, CSOs, Mental Health Research Network, National Health Service, NHS, England

## Abstract

**Background:**

Recruitment to trials is complex and often protracted; selection bias may compromise generalisability. In the mental health field (as elsewhere), diverse factors have been described as hindering researcher access to potential participants and various strategies have been proposed to overcome barriers. However, the extent to which various influences identified in the literature are operational across mental health settings in England has not been systematically examined.

**Methods:**

A cross-sectional, online survey of clinical studies officers employed by the Mental Health Research Network in England to recruit to trials from National Health Service mental health services. The bespoke questionnaire invited participants to report exposure to specified influences on recruitment, the perceived impact of these on access to potential participants, and to describe additional positive or negative influences on recruitment. Analysis employed descriptive statistics, the framework approach and triangulation of data.

**Results:**

Questionnaires were returned by 98 (58%) of 170 clinical studies officers who reported diverse experience. Data demonstrated a disjunction between policy and practice. While the particulars of trial design and various marketing and communication strategies could influence recruitment, consensus was that the culture of NHS mental health services is not conducive to research. Since financial rewards for recruitment paid to Trusts and feedback about studies seldom reaching frontline services, clinicians were described as distanced from research. Facing continual service change and demanding clinical workloads, clinicians generally did not prioritise recruitment activities. Incentives to trial participants had variable impact on access but recruitment could be enhanced by engagement of senior investigators and integrating referral with routine practice. Comprehensive, robust feasibility studies and reciprocity between researchers and clinicians were considered crucial to successful recruitment.

**Conclusions:**

In the mental health context, researcher access to potential trial participants is multiply influenced. Gatekeeping clinicians are faced with competing priorities and resources constrain research activity. It seems that environmental adjustment predicated on equitable resource allocation is needed if clinicians in NHS mental health services are to fully support the conduct of randomised controlled trials. Whilst cultural transformation, requiring changes in assumptions and values, is complex, our findings suggest that attention to practical matters can support this and highlight issues requiring careful consideration.

## Background

Archie Cochrane’s claim in 1972 that the importance of the randomised controlled trial (RCT) could not be exaggerated [1] was prophetic. With the rise of evidence-based approaches which now scaffold health care decision-making internationally, his view that evidence from trials was key to a rational health service has become normative. RCTs are conducted to examine the efficacy and effectiveness of diverse health technologies in achieving desired outcomes in diverse populations. Researchers design elegant trials, with due attention to statistical power and sample definition to facilitate generalizability of evidence. However, recruitment of the necessary sample, recognised as the single most important aspect of a successful trial [2], is often problematic [3,4]. When recruitment is protracted and/or insufficient to achieve desired power and samples are restricted to sub-sets of the target population, the return on large-scale investment in research and the generalizability of RCT evidence are compromised. Whilst of concern across health care, our interest is in the mental health context.

Extant literature indicates that the difficulties related to recruiting to mental health trials are complex. Barriers to access to potential participants have been located within target populations [3], trial designs and organisations [3-5], with the attitudes and knowledge of clinicians who mediate – or ‘gatekeep’ [6] – access to potential participants commonly cited as a key influence [5]. In this area generic challenges to research are potentially compounded by the perceived ‘vulnerability’ of potential participants. As gatekeepers, clinicians may seek to protect people for whom they provide care from the perceived burden of research participation and/or an intervention perceived to be futile for an intractable condition [3,4]. Symptom profiles also influence referral to studies [7] and sub-groups of the population are commonly underrepresented [8].

An emerging literature describes models, strategies and activities which can be applied to overcome barriers and promote recruitment [5,9,10] in various settings. Limited research conducted to date demonstrates that some of these, including reduction in the work associated with referral to RCTs, provision of research training and protected research time, show promise [9] but many remain untested.

With evidence to date primarily case study based, the extent to which various influences on recruitment to trials are operational in National Health Service (NHS) mental health settings is unknown. There has also been no systematic examination of the use and effectiveness of recruitment-promoting strategies. The absence of such information hinders development of a strategic approach to optimising recruitment to RCTs in mental health, undermining achievement of the governments’ commitment to making the NHS a research oriented, innovative organization [11]. With a view to supporting development of study design and recruitment strategies pertinent to the mental health context, we aimed to address this knowledge gap.

## Method

To achieve this aim we conducted a cross-sectional, online survey of researchers employed to recruit trial participants from NHS mental health services. Specifically in relation to researcher access to potential participants in NHS mental health services, our objectives were to describe:

i. To describe the extent to which influences on recruitment identified in the literature were encountered

ii. To describe the perceived impact of these factors on recruitment; and

iii. To identify additional factors perceived to influence recruitment.

A favorable ethical opinion was obtained from the North East UK Research Ethics Committee (reference number: 12/NE/0104) before data collection commenced.

### Materials

#### *Survey instrument*

The bespoke questionnaire was iteratively developed by the authors to address the study objectives. We began by drafting a pilot questionnaire, informed by a non-systematic but extensive review of the literature and the authors’ experiences as researchers. This questionnaire asked respondents to report exposure to factors reported to promote or hinder recruitment and rate the impact on access using a seven point scale - strongly negative to strongly positive. This draft was piloted with a convenience sample of 28 research assistants recruited from amongst participants in an in-service training day sponsored by the UK Mental Health Research Network (MHRN; see below). Pilot data and discussion with participants supported restructure of the questionnaire (specifically separation of reporting of exposure and impact and rescaling responses). The revision was trialled sequentially with another convenience sample of ten mental health researchers employed in the authors’ university departments with feedback used to improve comprehensibility. A penultimate version entered onto Survey Monkey [11] was tested for functionality by this same group of researchers. The final questionnaire (available on request) included an introduction, and open and closed questions in three sections. The introduction explained the purpose of the survey and defined ‘gatekeeping’ as allowing or denying a person’s access to a potential research participant. Sections one and two comprised the same list of 29 factors influencing researcher access to participants (see Table 1). These factors related to context, study design, intervention, study marketing and recruitment strategies. In section one, participants were asked to indicate whether they had ever encountered the identified factor while recruiting to trials and, if so, how often (selecting from ‘never’, ‘occasionally’, ‘commonly’ or ‘almost always’). In section two, they were asked to rate the impact of each of these factors on access to potential trial participants by selecting from ‘positive’, ‘neutral’, ‘negative’ or ‘don’t know’. Respondents were required to make a response to each item to continue questionnaire completion. Open questions at the end of each section invited participants to expand on responses and provide further information about other factors which, in their experience, influenced recruitment. Section three sought demographic data, but no identifying information; completion was anonymous.

**Table 1:** Prevalence of experience and reported impact of different factors on access to potential participants as reported by 98 CSOs

		**A. Has participant experience of factor?**	**B. Participants assessment of the influence of factor on access to participants**		
		**Valid responses = 98 (unless stated)**	**Enhances access**	**Makes no difference**	**Inhibits access**
		**N (%)**	**N (%)**	**N (%)**	**N (%)**
	**Site management position re. trial**				
1	Gatekeepers refer to the NHS commitment to patient participation in research	**41 (41.8)**	*68 (69.4)*	*28 (28.6)*	*2 (2.0)*
2	Gatekeepers have protected time to undertake research activity	**55 (56.1)**	*81 (82.7)*	*8 (8.2)*	*9 (9.2)*
3	Service management proactively endorse the trial and promote referral to the study	**77 (78.6)**	*93 (94.9)*	*4 (4.1)*	*1 (1.0)*
4	Managers instruct gatekeepers to refer potential participants	**76 (77.6)**	*83 (84.7)*	*9 (9.2)*	*6 (6.1)*
5	An identified member of the gatekeeping team is responsible for facilitating referral	**76 (77.6)**	*80 (81.6)*	*15 (15.3)*	*3 (3.1)*
	**Trial design**				
6	Concurrent recruitment to multiple trials at research site	**89/97 (90.8)**	*11 (11.3)*	*24 (24.7)*	*62 (63.9)*
7	Trial design is non-simple	**78/97 (79.6)**	*0 (0)*	*26 (26.8)*	*71 (73.2)*
8	Gatekeepers are masked to treatment allocation	**88/97 (89.8)**	*5 (5.2)*	*68 (70.1)*	*24 (24.7)*
	**Site characteristics**				
9	Site is undergoing substantial change such as reconfiguration or restructuring	**92 (93.9)**	*0 (0)*	*3 (3.1)*	*95 (96.9)*
	**Relationship between recruitment and clinic procedures**				
10	Trial participation requires ongoing involvement of the gatekeeper (e.g. enabling attendance at an intervention/completing outcome measures)	**84 (85.7)**	*12 (12.2)*	*10 (10.2)*	*76 (77.6)*
11	Referral to the trial requires gatekeepers to complete questionnaires or an interview about themselves	**45 (45.9)**	*3 (3.1)*	*17 (17.3)*	*78 (79.6)*
12	Assessment of eligibility is integrated with routine clinical processes (e.g. case review)	**68 (69.4)**	*90 (91.8)*	*6 (6.1)*	*2 (2.0)*
13	Referral requires gatekeepers to complete questionnaires or an interview about trial participants	**80 (81.6)**	*6 (6.1)*	*12 (12.2)*	*80 (81.6)*
	**Characteristics of the trial intervention**				
14	The trial intervention is only accessible through referral to the trial	**95 (96.9)**	*52 (53.1)*	*25 (25.5)*	*21 (21.4)*
15	Gatekeepers hold views about the effectiveness and/or appropriateness of the trial intervention (gatekeeper would not accept that equipoise exists)	**93/97 (94.9)**	*7 (7.2)*	*6 (6.2)*	*84 (86.6)*
	**Trial marketing**				
16	Trial investigators have a structured approach to promoting the trial to gatekeepers	**81 (82.7)**	*76 (77.6)*	*17 (17.3)*	*5 (5.1)*
17	A senior member (e.g. principal investigator) of the research team meets with gatekeepers to promote the trial	**74 (75.5)**	*89 (90.8)*	*8 (8.2)*	*1 (1.0)*
18	Gatekeepers receive structured updates (e.g. regular newsletters/formal feedback at meetings) about recruitment progress	**90 (91.8)**	*82 (83.7)*	*15 (15.3)*	*1 (1.0)*
19	The trial is marketed directly to the target population (with a view to potential participants seeking referral)	**90 (91.8)**	*87 (88.8)*	*10 (10.2)*	*1 (1.0)*
	**Service user involvement**				
20	Members of the target population (those potentially affected by trial outcomes) promote referral to the trial, with gatekeepers	**39/97 (39.8)**	*87 (89.7)*	*10 (10.3)*	*0 (0)*
21	Gatekeepers are advised that the target population has been involved in the trial design and/or implementation	**79 (80.6)**	*69 (70.4)*	*28 (28.6)*	*0 (0)*
	**Clinician attitudes**				
22	Researchers share the professional background of the gatekeeper	**78 (79.6)**	*58 (59.2)*	*39 (39.8)*	*1 (1.0)*
23	Gatekeepers express a personal interest in research	**93 (94.9)**	*94 (95.9)*	*2 (2.0)*	*2 (2.0)*
24	Gatekeepers base referral on criteria other than those specified in the trial protocol (i.e. they make referrals on the basis of their opinion about suitability)	**85/94 (86.7)**	*11 (11.7)*	*12 (12.8)*	*72 (76.6)*
25	Gatekeepers believe trial participation will negatively affect their relationship with potential participants	**76 (77.6)**	*1 (1.0)*	*5 (5.1)*	*92 (93.9)*
	**Incentives/motivational interventions**				
26	Incentives (e.g. chocolates/prize draw) are provided to gatekeepers for the referral of potential participants	**54 (55.1)**	*70 (71.4)*	*26 (26.5)*	*2 (2.0)*
27	Researchers encourage competition between study sites in relation to recruitment	**86 (87.8)**	*48 (49.0)*	*47 (48.0)*	*3 (3.1)*
28	Researchers are set specific recruitment targets which are monitored by their management	**96 (98.0)**	*54 (55.1)*	*40 (40.8)*	*4 (4.1)*
29	Site recruitment targets (number of participants and/or time frames) are agreed with gatekeepers	**75 (76.5)**	*57 (58.2)*	*37 (37.8)*	*4 (4.1)*

#### *Study sample and recruitment*

The sample comprised clinical studies officers (CSOs) employed by the MHRN. The MHRN is one of six topic-specific networks established in 2003 as part of the National Institute of Health Research (NIHR), to facilitate research within the NHS. CSOs at each of eight locality-based MHRN hubs support and conduct recruitment into publicly and privately funded trials at NHS Trusts within the hub’s geographically defined area. While the specific roles and activities of CSOs may differ dependent on study or site, in general CSOs are charged with working collaboratively with clinicians to identify people who are potentially eligible for trial participation. Once access to potential participants is achieved CSOs verify eligibility and undertake consent and study assessment procedures. CSOs are thus well positioned to describe factors influencing access to participants.

After securing approval of the MHRN Operational Managers Committee in January 2013, the manager of each hub was emailed a study synopsis, participant information sheet and a link to the online survey. Managers were asked to forward the email and attachments to all CSOs employed by the hub. Managers were sent reminder emails for distribution to CSOs two and four weeks after initial contact. The survey link was closed at the end of February 2013.

#### *Analysis*

Data were downloaded from Survey Monkey [12] to SPSS version 20 [13] for analysis. Descriptive statistics were used to profile participants and quantify responses regarding experience of influences on recruitment and perceptions of impact. Frequency data were tabulated for categorical variables and means (M) and standard deviations (SD) calculated for continuous data. Free text responses were entered onto Microsoft Word and subject to thematic analysis using the framework approach [14] which supported examination of responses relevant to research objectives and matters arising in respondents’ comments. The first step in the qualitative analysis was to populate an initial frame comprising cells representing ‘research objective by participant’ with data from open questions, with multiple allocations possible. New cells were created as required to accommodate data. Once all data were allocated to cells, constant comparison and analytic questioning were employed to discern patterns and exceptions in the data. The frame was iteratively developed to support exploration of data-based questions generated as analysis progressed (for example, to examine the ways ‘culture’ impacted recruitment) and relationships between the themes. Finally quantitative and qualitative findings were triangulated to construct the narrative account presented below, which includes verbatim quotations in “*italics”*. Analysis was supported throughout by individual and team reflexivity as developing findings were subject to intellectual and critical scrutiny [15]. This scrutiny was underpinned by a pragmatic commitment to developing findings which could be usefully applied to real world problems.

## Results

### Participants

Data were provided by 98 CSOs, (57.6% of the 170 CSOs employed in all MHRN hubs at the time of dissemination). (Unless stated the denominator in all frequency analyses are the full 98 respondents). Demographic data presented in Table 2, show that respondents were aged between 23 and 58 years (M = 35.3, SD = 9.4). They were predominately female (80, 81.6%) and White British (75, 76.7%). Ninety (91.8%) were tertiary educated with 43 (43.9%) having Masters (39; 39.8%) or PhD degrees (4; 4.1%). Participants reported qualifications in nursing (24; 24.5%), psychology (31; 31.6%), allied health (11; 11.2%), and physical sciences (4; 4.1%), with several reporting multiple professional and/or academic qualifications. Experience recruiting to trials varied widely from ‘just started’ to 20 years (M = 4.3 years, SD = 4.3). Participants had recruited to an average of six trials (SD = 4.7) from nine (SD = 9.1) sites, often concurrently recruiting from multiple populations and settings. Nearly all (87; 88.8%) had recruited from community mental health services, a third from residential services (33; 33.7) and almost one quarter (22; 22.4%) from forensic or secure settings.

**Table 2:** Demographic details and research experience of 98 CSOs

**Variable**	**Response**	**N (%)**
**Gender**	Male	14 (14.3)
	Female	80 (80.6)
	Missing	4 (4.1)
**Age**	Years (Mean, SD)	35.3 (9.4)
**Ethnicity**	White British/White Other	81 (82.7)
	Asian/Asian British	10 (10.2)
	Black/Black British African	1 (1.0)
	Black/Black British Caribbean	1 (1.0)
	Other	1 (1.0)
	Missing	4 (4.1)
**Highest academic qualification**	Diploma	5 (5.1)
	Bachelor’s degree	42 (42.8)
	Masters degree	39 (39.8)
	PhD	4 (4.1)
	Missing	8 (8.2)
**Professional qualifications**	Psychology	31 (31.6)
	Nursing	24 (24.5)
	Allied health	11 (11.2)
	Physical sciences	4 (4.1)
**From which populations have you recruited to RCTs?**	Children (aged <13)	33 (33.7)
	Adolescents (aged 13-18)	50 (51.0)
	Adults (aged 18-65)	87 (88.8)
	Older adults (aged >65)	49 (50.0)
	Severe mental illness	75 (76.5)
	Eating disorders	25 (25.5)
	Common mental health problems	67 (68.4)
	Drug & alcohol misuse problems	54 (55.1)
	Gambling problems	4 (4.1)
**From which settings have you recruited to RCTs?**	Primary care	37 (37.8)
	Community mental health services	87 (88.8)
	Inpatient units	59 (60.2)
	Residential services	33 (33.7)
	Secure/forensic services	22 (22.4)
**Years of experience in research**	Mean (SD)	4.3 (4.3)
**Years of experience recruiting to RCTs**	Mean (SD)	3.4 (3.4)
**Number of RCTs recruited to**	Mean (SD)	5.8 (4.7)
**Number of sites recruited from**	Mean (SD)	9.1 (9.1)

In combination, quantitative and free-text data provided insight to the complexities of recruiting to trials in NHS mental health settings. Responses to sections one and two of the questionnaire are summarised in Table 1. As shown, responses indicated that diverse factors were influencing recruitment in NHS mental health services. Respondents generally agreed about whether particular factors promoted or hindered access to potential participants. As shown in Table 3 most factors thought to impact positively, and some of those thought to impact negatively on access to potential participants, were commonly experienced by CSOs.

**Table 3:** Distribution of factors influencing recruitment by reported prevalence and impact

	**Very commonly reported (80% +)**	**Reported by majority (50-79%)**	**Reported by minority (<50%)**
**Strong consensus (>80%) that factor enhances access to potential participants**	**23.** Gatekeepers express interest in research	**17.** A senior member of the research team meets gatekeepers to promote the trial.	**20.** Members of the target population (those potentially affected by trial outcomes) promote referral to the trial with gatekeepers.
	**18.** Gatekeepers receive structured updates about recruitment progress	**4.** Managers instruct gatekeepers to refer participants.	
	**19.** The trial is marketed directly to the target population	**5.** An identified member of the gatekeeping team is responsible for facilitating referral.	
		**3.** Site management proactively endorses trial referral.	
		**12.** Assessment of eligibility is integrated with routine clinical practice.	
		**2.** Gatekeepers have protected time for research.	
**Majority (50-79%) felt that factor enhances access to potential participants**	**14.** The intervention is only accessible through the trial.	**29.** Site recruitment targets (number of participants and/or time frames) are agreed with gatekeepers.	**1.** Gatekeepers refer to the NHS commitment to patient participation in research.
	**28.** Researchers set recruitment targets.	**22.** Researchers share the professional background of the gatekeeper.	
	**16.** Trial investigators have a structured approach to promoting the trial.	**26.** Incentives (e.g. chocolates/prize draw) are provided to gatekeepers for the referral of potential participants.	
	**21.** Gatekeepers are advised that the target population has been involved in trial design		
**Majority (>50%) reported factor made no difference**	**27.** Researchers encourage competition between study sites in relation to recruitment		
	**8.** Gatekeepers are masked to treatment allocation		
**Majority (50-79%) felt that factor inhibits access to potential participants**	**6.** Concurrent recruitment to multiple trials is taking place from individual sites.		**11.** Referral to the trial requires gatekeepers to complete questionnaires or an interview about themselves.
	**24.** Gatekeepers base referral on criteria other than those specified in the trial protocol.		
	**15.** Gatekeepers hold views about the effectiveness/appropriateness of the intervention.	**7.** Trial design is non-simple.	
**Strong consensus (>80%) that factor inhibits access to potential participants**			
	**9.** Site is undergoing substantial organisational restructuring/change.	**25.** Gatekeepers believe trial participation will negatively affect their relationship with potential participants.	
	**10.** Trial participation requires ongoing involvement of the gatekeeper.		
	**13.** Referral requires gatekeepers to complete questionnaires or an interview about service users.		

Free-text responses provided by 86 respondents (87.8%) expanded on and qualified responses, ‘explaining’ findings and describing additional influences on recruitment. Differentiating recruitment in mental health from other services, CSOs also wrote of contextual variability within the sector, identifying cultural, structural, organisational, trial, setting and interpersonal factors as influencing access to potential participants. They emphasised the need for strategic and tactical flexibility if access is to be optimised.

### Culture and gatekeeping

The broad consensus amongst respondents (94, 95.9%) was that recruitment potential was optimised when clinicians with a personal interest in research (item 23) were working within research-friendly cultures. However, such circumstances were described as the exception rather than the rule. Endorsement of the NHS commitment to service user participation in research (item 1), described as enhancing access, was experienced by fewer than half (41, 41.8%) of participants. Clinicians were often described as “*too busy*” to undertake recruitment (and other research) activities. CSOs observed that research was considered *“extra work*” rather than a core responsibility. Hence, gatekeepers often “*forgot to ask”* potential participants about studies.

Gatekeepers are busy with long lists of responsibilities; research comes way down that list. The lack of a research culture within the NHS is the biggest barrier. If staff don't see research as an opportunity or relevant or are intimidated by research, they won't promote it. It's not about specific staff; it's about the way a team, the service and the organisation perceive research.

More than three-quarters of CSOs (72/94, 76.6%) reported that gatekeepers applying non-study criteria to referral had a detrimental impact on referral into trials (item 24). Criteria reported to be used by clinicians included the likelihood of the service user accepting the invitation, the perceived benefit and the predicted impact on their clinical relationship. Many CSOs described gatekeepers as paternalistic and over-protective of service users, observing that service users’ autonomy was undermined when referrals were based on *“opinions about suitability or the work associated with referral”*. Whilst acknowledging clinicians’ expert position and service user advocacy roles, respondents argued for service users’ rights to make decisions *“for themselves*”.

Just as they should be involved in decisions about their care, service users should have a choice about research.

Perceived clinician negativity and ambivalence were linked to structural and organisational issues. CSOs wrote that clinicians were distanced from research and that research-related activity often went unrewarded in services. They linked this with disjunctions between espoused NHS commitment to research, research and development (R&D) departments responsible for oversight of research within Trusts and the clinical services from which they attempted to recruit. Consensus was that proactive and pragmatic R&D departments and/or service managers, and consistency of research message within organisations (items 3 [93, 94.9%] and 4 [83, 84.7%]), could enhance recruitment potential.

Unless senior managers endorse research, the 'it’s not for us' attitude prevails and it is an uphill struggle to get PIs [Principal Investigators] and clinicians interested in studies.

CSOs wrote that the apparent failure to translate financial incentives paid to Trusts for ‘accrual’ of research participants into benefits for clinical teams reinforced clinician scepticism.

What incentives do clinical teams get from their Trust (rather than the odd packet of chocolate biscuits from me)?

Within such circumstances, CSOs appreciated gatekeepers’ perception of research as burdensome, rather than as a resource (i.e., funding source, enabling access to an intervention, source of practice-relevant knowledge). This already problematic situation was compounded, according to respondents, by the restructuring of services (item 9) which was a factor encountered by most respondents (95, 96.9%) of whom almost all felt it inhibited access (92, 93.9%). The negative impact on recruitment was attributed to demoralisation of clinicians who experienced job insecurity and lacked the motivation and cognitive resources needed to undertake research activity.

### Study team, trial design and gatekeeping

Data indicated that attention to the impact upon clinicians of study design and the procedures for referral into a trial could strongly influence access. The activities of the study team including various marketing strategies (e.g. its promotion by investigators, feedback to clinicians on recruitment), and publicised service user involvement in the research were also considered influential. Fundamentally, however, CSOs argued strongly that rigorously conducted feasibility studies which realistically assessed recruitment potential were crucial to success of a trial. Such studies would move beyond numbers of potential participants enrolled at a service to examination of stakeholder interest in research and capacity to support recruitment; service stability and culture should be examined. CSOs urged research teams to engage early in the planning of a study with the MHRN, clinical teams and service user representatives.

Ask individuals who regularly work with studies and with teams about potential pitfalls or strategic knowledge at the development stage - by the time the study is approved it is often too late to do anything about it.

The majority of CSOs (76, 77.6%) proposed that such engagement would inform a contextually relevant, structured approach to recruitment (item 16).

In relation to study design, most CSOs thought non-simple (i.e., complex) trial designs impeded recruitment (item 7) (71/97, 73.2%). Some aspects of trial designs were described as difficult to communicate and gatekeepers were seen as less likely to refer when they did not understand exactly what would happen to potential participants during a study.

Integrity of the study, congruence of the research question with service philosophy and trial intervention with local practice were commonly described as enhancing recruitment.

Few care co-ordinators are interested in telling patients about pharmaceutical trials, possibly because they don’t see prescribing medications as their role.

Asserting that gatekeepers refer service users to studies they consider worthwhile and relevant, CSOs observed that involvement of members of the target population in study design (item 20; 87/97, 89.7%) and promotion (item 21; 69, 70.4%), and site visits by senior investigators (item 17; 89, 90.8%) each enhanced recruitment by building credibility amongst clinicians. Several CSOs reported finding recruitment easier when trial participation facilitated access to a desired intervention and/or could be viewed as part of the treatment package provided by the service. Conversely, recruitment was hindered when participation made accessing a readily available treatment more burdensome, or clinicians held strong views about the intervention being tested (item 15) (84, 86.6%). Masking gatekeeping clinicians to treatment allocation (item 8), however, was regarded by the majority (68, 70.1%) as having little impact on recruitment.

The more work involved, the less likely they are to help with recruitment.

Perceived or actual demands of study activities on gatekeepers and participants were described as impacting on recruitment. Whereas integrating assessment of eligibility with routine clinical processes (item 12) was viewed as enhancing recruitment by the vast majority (90, 91.8%) of CSOs, gatekeepers were described as less likely to refer when they were required to complete questionnaires about their own practice (item 11; 78, 79.6%) or potential participants (item 13; 80, 81.6%) or if participation required them to provide ongoing clinical support (item 10; 76, 77.6%). However, CSOs considered clinician support crucial to gathering needed data and noted that information gathered was better quality when clinicians completed assessment forms. With protection of potential participants high on clinicians’ agendas, recruitment potential was described as compromised when participation involved complex and multiple assessments. Conversely, gatekeepers were considered more likely to refer when trial interventions could be carried out in a manner consistent with routine practice, meaning minimal inconvenience to participants and fewer demands upon the time of clinical staff involved.

CSOs regarded active involvement of a research assistant (RA) employed by the study team as critical to recruitment success. Noting that CSOs could be required to be aware of as many as 100 trials while actively recruiting to many (number not specified) at a given time, respondents suggested that the study RA could plan/coordinate promotion and recruitment activities, ensuring that CSO energies were invested appropriately.

### Communication and study materials

Simple, attractive study materials and easy to understand practical presentations which engaged clinicians were considered crucial. Provision of a *“really basic summary* – *a couple of sentences describing the study, then bullet point main inclusion/exclusion criteria”* was described as minimising burden and thus the risk of ‘screening out’ inappropriately.

Teams prefer an easy read; they want to know what is expected, not a mundane presentation that they probably don't understand but won’t ask questions about. They prefer to see what they have to do in practice.

The vast majority (82, 83.7%) agreed that keeping clinicians informed about study and recruitment progress (item 18) enhanced recruitment. Various communication mechanisms, including newsletters and presentations at meetings were proposed with ’fit’ with circumstance and relationship considered important determinants of approach. They also emphasised the importance of making study findings available to recruitment sites as they became available. As noted by one respondent, inability to access study results, perhaps because they are “*behind a ‘pay wall’”*, served to distance gatekeepers from research, further discouraging engagement.

### Incentives

CSOs expressed divergent opinions regarding the impact of incentives for participants in recruitment. Concern was expressed that clinicians who considered incentives coercive inducement were reluctant to refer. However, more commonly, CSOs’ reported that financial incentives enhanced referral. The provision of incentives was described as having an unintended impact as well:

Financial incentives for commercial studies significantly drives recruitment, preventing referral to studies without the same incentives.

Provision of incentives to gatekeepers (item 26) was endorsed as enhancing recruitment by almost three quarters (70, 71.4%) of respondents.

Never take support for granted and remember that as researchers you’re guests in the clinical area. Always show respect to staff, patients and carers. Cakes/chocolates are appropriate to thank staff for supporting research at busy times and at festival times.

Several CSOs suggested going beyond gifts of chocolate and cake which were commonly described as being helpful in encouraging clinicians’ engagement. CSOs could ‘give back’, they proposed, by providing or facilitating access to research-related training and investigators could offer publication involvement to motivate gatekeeper engagement.

Free text expanded on the divergence of opinion evident in response to item 6 which assessed the way in which concurrent recruitment to multiple trials from a given site could affect recruitment. Recruitment could be made more difficult if gatekeepers were overwhelmed by requests for referrals or when CSOs and studies were in competition for a particular population group. Alternatively, some respondents thought that recruitment could be enhanced when potential participants who were ineligible for one study - but keen to participate in research - could be referred to another. The increased presence at a site when CSOs were recruiting concurrently to multiple studies was also seen as supporting establishment of collaborative relationships, conducive to recruitment.

### Enhancing recruitment: what can be done?

Development of a research receptive culture supporting a critical mass of supportive gatekeepers was described as critical to optimising recruitment. This, according to respondents, would require investment (perhaps using accrual funding) in training of clinicians and protected research time.

More work needs to be done to free up time of gatekeepers for involvement with research and more education needs to be provided by study teams to gatekeepers to generate interest.

CSOs proposed that academic departments and individual investigators had responsibility for developing the needed research culture. Noting that gatekeepers often felt ‘used’ by researchers, they argued that research would only gain credibility when researchers were seen to contribute to building capacity within services.

### Relationships and recognition

At the interface between researchers and clinicians, relationship was widely regarded as the key to effective recruitment. CSOs stressed the importance of establishing good working relationships with gatekeepers including/especially service administrative staff *before* recruiting to a study commenced and maintaining good-will over time.

If a good link is made from site initiation and sustained, recruitment outcomes are on target 9/10 times more than if research staff drift.

Relationships facilitative of recruitment were described as grounded in role clarity, respect for others’ responsibilities and reciprocity. CSOs wrote of the importance of acknowledging challenges faced by clinicians and their efforts to support research.

Communicating the right (relevant) information to the right people, often repeatedly, was seen as critical to ensuring roles and responsibilities were clear and encouraging engagement.

High visibility was commonly described as facilitating the formation and maintenance of the necessary relationships. In this regard, CSOs reported that ‘having a desk’ within a clinical team and regularly attending meetings enabled development of trust and a sense of shared responsibility for the success of trials. Some promoted the idea that CSOs should be employed by, and located within, Trusts so that they ‘really were one of the team’.

### Direct access – cutting out the middle man

Many CSOs wrote that “*direct access”* to potential participants and *immediacy of contact* following referral would enable timely recruitment. CSOs considered direct access respectful of potential participants’ autonomy because gatekeepers were not making decisions on their behalf. They wrote that enrolment was more likely when potential participants could “*see that research has a friendly face”* and information could be provided without delay. Allowing recruitment in waiting rooms and establishing databases of service users willing to be approached about studies were proposed as enabling respectful access.

## Discussion

Our cross-sectional survey of CSOs has illuminated the complexities inherent in the task of recruiting trial participants from NHS mental health services. Collectively, findings confirm and consolidate findings from studies based in particular trials [3,9,10], pointing to a disjunction between policy and practice. Despite government declaring a commitment to making research core business of the NHS [11], this was not seen by respondents in our study to be facilitated at the clinical level. Rather than being enabled to enact the pledge in the NHS constitution to inform patients of studies in which they may be eligible to participate [16], the majority of mental health clinicians charged with gatekeeping access are struggling to cope with competing demands and unable to prioritise research. Rather than researchers and clinicians being allies sharing a purposeful approach to development of evidence, our findings are suggestive of a culture resistant to research. This is likely grounded in contextual complexities (including physical and emotional distance between R&D departments and the clinical ‘frontline’, and the burden and demands of clinical work. The reported resistance may in part be grounded in the redirection of funding from clinical services to establish the NIHR to increase research capacity within services. It may also reflect a lack of fit between services and clinicians and the approaches taken by the MHRN. Moreover, given recruitment is essentially a social activity, with success dependent in part on interpersonal connection between recruiting researchers and clinicians [5], it may well be that some of the challenges described reflect personal rather than systemic concerns. The key message arising from this study, however, is that rapprochement between the NHS R&D and clinical service factions and academia is needed to support clinicians to participate fully in research activity. Whilst cultural transformation, requiring changes in assumptions and values, is complex, our findings suggest that attention to practical matters can support this and highlight issues requiring careful consideration. Before considering these we note study limitations which must be borne in mind when assessing the strength of our evidence.

The nature of the study (cross sectional) and its application (construction, sampling and response rate) mean that we cannot know how representative the picture constructed from our data is and cannot make any causal assertions. Our study examined only one perspective of a complex phenomena (i.e. gatekeepers did not have a ‘right of reply’) and findings are necessarily constrained chronologically and geographically. They are inherently shaped by the characteristics and perspectives of participant CSOs. It is entirely possible that other CSOs - perhaps working on different trials - or another sample (perhaps of researchers based in study teams) might have different experiences and views which may have challenged or enriched our findings. NHS sites not covered by the MHRN differ in ways which substantively affect recruitment to trials. Moreover, as with any qualitative analysis, our representation of respondents’ views expressed in free-text is vulnerable to claims of bias; we acknowledge that others may have made alternate interpretations. Confidence in our findings is strengthened, however, by the breadth and depth of experience of the nearly 60% of eligible CSOs who responded and the wealth of contextualizing data provided. Triangulating of quantitative and qualitative responses has enabled development of a credible account of recruitment challenges and opportunities giving rise to recommendations below.

Reciprocity, relevance, respect and realistic feasibility studies are fundamental to ensuring that recruitment to trials is to be efficient and clinician-related selection bias is minimized. Rather than feeling ‘put upon’ by disinterested researchers, gatekeepers should be involved in the research process from the beginning and have their contributions valued. It behoves researchers, CSOs and the MHRN to work actively to support clinician and service engagement and minimise the burden of research activity. In relation to relevance, the onus is on investigators and NHS leaders to engage collaboratively with clinicians to develop research questions and design studies which have face validity and will garner support. Resources are critical. Because the absence of dedicated resources (such as clinical time) or return on investment (such as allocation of accrual funding to team training) not only constrains the capacity of clinicians to undertake research activity but undermines belief in policy and sense that their roles are respected, research resources must be seen to make a difference. At a practical level, respect and reciprocity can be promoted by effective communication. Ensuring that the right information reaches the right people in a timely manner, and that clinicians are provided with progress reports and study findings, is essential. Offers of publication involvement, though potentially encouraging activity, should be made only as they align with ethics of authorship and the mixed messages around incentives mean that these should carefully be considered on a case-by-case basis.

While we set out to explore recruitment to trials in mental health, we note that the recommendations grounded in our data could be seen as generic in that they could apply across health care domains. Determining which factors, if any, influencing recruitment are peculiar to ‘mental health’ would require a comparative approach which our study did not have, but which we would encourage other investigators to adopt. However, the elusiveness of clinical gains amongst many mental health service users, the fragility of these gains given the high rates of relapse and the reality that when gains are achieved they often require treatment over the long term, may be one key influence. It is possible that this generates a conservative and cautious approach to gatekeeping amongst mental health workers who when invited to judge the potential positive and negative consequences of participation may see the scales tipped to often towards the negative.

## Conclusions

Further research is needed to explicate the complex relationships between researchers and clinicians to support development of the necessary alliances. It seems particularly important that clinicians’ voices are heard. Ultimately, there are no simple solutions. If Cochrane's goal of equitable access to interventions shown to be effective in properly designed evaluations is to be achieved, the cultural foundation upon which gatekeepers scaffold evidence construction must be robust.

## Abbreviations

CSO: Clinical studies officer; MHRN: Mental Health Research Network; NIHR: National Institute for Health Research; NHS: National Health Service; RCT: Randomised controlled trial; UK: United Kingdom.

## Competing interests

All authors have completed the Unified Competing Interest form at http://www.icmje.org/coi_disclosure.pdf (available on request from the corresponding author) and declare that RB, TW, DW, DP and SP have no competing interests.

## Authors’ contributions

SP conceptualized the study and drafted the protocol, collaborated in design of the questionnaire, analysis of data and interpretation. RB collaborated in design of the questionnaire, interpretation of data and writing of the manuscript. TW collaborated in design of the questionnaire, analysis and interpretation of data and writing of the manuscript. DP and DW contributed to design of the questionnaire and writing of the manuscript. SP is guarantor. All authors read and approved the final manuscript.

## Authors’ information

SP and RB are both clinical psychologists. TW is a medical sociologist and DP is a health services researcher. DW has qualifications in psychology and has been working within clinical research for eight years. All are actively engaged in research requiring negotiation of gatekeeping.

## Pre-publication history

The pre-publication history for this paper can be accessed here:

http://www.biomedcentral.com/1471-2288/14/23/prepub
